# Digital Transformation in Critical Care: Implications for Quality of Care, Infection Control, and Clinical Outcomes

**DOI:** 10.3390/jcm14248964

**Published:** 2025-12-18

**Authors:** Daiana Toma, Laura Andreea Ghenciu, Ovidiu Horea Bedreag, Adelina Băloi, Carmen Alina Gizea, Stelian Adrian Rițiu, Emil Robert Stoicescu, Claudiu Rafael Bârsac, Marius Păpurică, Alexandru Rogobete, Dorel Săndesc

**Affiliations:** 1 Anaesthesia and Intensive Care Research Center, Faculty of Medicine, “Victor Babes” University of Medicine and Pharmacy, 300041 Timisoara, Romania; daiana.toma@umft.ro (D.T.); papurica.marius@umft.ro (M.P.); alexandru.rogobete@umft.ro (A.R.); sandesc.dorel@umft.ro (D.S.); 2 Doctoral School, “Victor Babes” University of Medicine and Pharmacy, 300041 Timisoara, Romania; stelian.ritiu@umft.ro (S.A.R.); claudiu.barsac@umft.ro (C.R.B.); 3 Discipline of Pathophysiology, Department of Functional Sciences, “Victor Babes” University of Medicine and Pharmacy, Square Eftimie Murgu 2, 300041 Timisoara, Romania; bolintineanu.laura@umft.ro; 4 Center for Translational Research and Systems Medicine, “Victor Babes” University of Medicine and Pharmacy, Square Eftimie Murgu 2, 300041 Timisoara, Romania; 5 County Emergency Hospital ‘Pius Brinzeu’ Timisoara, 300723 Timișoara, Romania; carmalig@yahoo.com; 6 Radiology and Medical Imaging University Clinic, “Victor Babes” University of Medicine and Pharmacy, Eftimie Murgu Square No. 2, 300041 Timisoara, Romania; stoicescu.emil@umft.ro; 7 Research Center for Medical Communication, “Victor Babes” University of Medicine and Pharmacy, Eftimie Murgu Square No. 2, 300041 Timisoara, Romania; 8 Research Center for Pharmaco-Toxicological Evaluations, “Victor Babes” University of Medicine and Pharmacy, Eftimie Murgu Square No. 2, 300041 Timisoara, Romania

**Keywords:** clinical decision support, critical care informatics, device utilization, digitalization, digital transformation, health information technology, intensive care unit, length of stay, nosocomial infection, patient data management system, workflow efficiency

## Abstract

**Background/Objectives:** Digitalization of intensive care units (ICUs) aims to enhance patient safety and efficiency through standardized documentation, real-time data integration, and clinical decision support. This study evaluated whether the implementation of a patient data management system (PDMS) was associated with improvements in quality of care, infection prevention, and patient outcomes in a trauma ICU. **Methods**: We conducted a single-center, retrospective, before–after cohort study comparing a pre-digitalization period (2021–2022) with a post-digitalization period (2025). Consecutive adult trauma ICU admissions were analyzed. The exposure was unit-wide adoption of a PDMS implemented in 2024. The primary outcome was ICU length of stay (LOS); secondary outcomes included ICU mortality, nosocomial infection rates (episodes per 1000 ICU-days), ventilation- and antibiotic-days, device utilization, and infection epidemiology. Prespecified sensitivity analyses were performed. **Results**: A total of 108 patients were included (43 pre- and 65 post-digitalization). Baseline characteristics were comparable between groups. Median ICU LOS decreased from 13.0 to 6.0 days (*p* = 0.02). Mortality declined from 18.6% to 6.2% (*p* = 0.06), and crude infection rates decreased from 42.2 to 30.8 per 1000 ICU-days (rate ratio 0.73; *p* = 0.28). Adjusted analyses showed no statistically significant differences for mortality (aOR 0.40; *p* = 0.45), infection rates (aIRR 0.88; *p* = 0.68), LOS (aRR 1.04; *p* = 0.87), ventilation-days (aRR 0.86; *p* = 0.65), or antibiotic-days (aRR 0.70; *p* = 0.30). Per-patient rates of ventilator-associated pneumonia and bloodstream infection were significantly lower after digitalization (both *p* = 0.04), and *Acinetobacter* spp. infections decreased markedly (7 to 0 cases; *p* = 0.001). Findings were consistent after exclusion of ICU stays < 24 h. **Conclusions**: ICU digitalization was associated with shorter unadjusted ICU stays and favorable trends in infection and mortality outcomes, though adjusted analyses were neutral. Larger multicenter studies incorporating device-day denominators and time-to-event analyses are needed to confirm the causal impact of digital transformation on ICU quality of care.

## 1. Introduction

Intensive care units (ICUs) are complex environments where critically ill patients require continuous monitoring, rapid clinical decision-making, and the coordinated use of advanced therapeutic interventions [1,2]. Despite significant advances in critical care medicine, ICUs remain highly resource-intensive settings, where outcomes are strongly influenced not only by the severity of illness but also by organizational efficiency, timely communication, and error prevention [3]. In this context, digitalization has emerged as a transformative force, reshaping the way information is collected, processed, and acted upon in critical care [4,5].

Nosocomial infections remain a major challenge in intensive care units, contributing substantially to morbidity, mortality, and prolonged hospitalization. ICU patients are especially prone to device-associated infections such as ventilator-associated pneumonia (VAP), central line-associated bloodstream infection (CLABSI), and catheter-associated urinary tract infection (CAUTI) because of invasive procedures, mechanical ventilation, and extended lengths of stay [6,7,8]. These infections frequently necessitate broad-spectrum antimicrobial therapy, resulting in disproportionately high antibiotic exposure compared with other hospital settings [9,10]. Excessive or inappropriate antibiotic use in the ICU promotes antimicrobial resistance, disrupts the patient microbiome, and increases the risk of drug-related toxicity [11,12]. Consequently, infection-prevention strategies and antimicrobial-stewardship programs have become essential components of ICU quality and safety initiatives. Digital systems can support these efforts by standardizing documentation, integrating real-time surveillance, and facilitating timely feedback on antibiotic prescribing and device use [13,14,15]. ICU mortality also remains high despite advances in critical care, with reported rates ranging from 10 to 30% depending on case mix and acuity, showing the need for systems that support timely clinical decisions and early recognition of deterioration; emerging evidence suggests that digital platforms may contribute to improved survival by enhancing situational awareness and reducing preventable adverse events [16,17,18].

Digital technologies in the ICU encompass a wide spectrum, ranging from electronic health records (EHRs) [19], computerized physician order entry [15], and clinical decision support systems [20], to real-time monitoring dashboards, automated alert systems, and tele-ICU platforms [21,22,23]. These innovations aim to improve data integration, reduce delays in diagnosis and treatment, and support clinicians in managing the overwhelming volume of patient information [24]. By streamlining workflows and facilitating evidence-based decisions, digitalization has the potential to reduce medical errors, optimize the use of invasive devices and antibiotics, and ultimately improve survival and recovery [14,25].

Recent studies suggest that the implementation of digital tools in critical care can impact both clinical and organizational outcomes. Benefits include reductions in medication errors, shorter ICU stays, better infection control, and enhanced resource allocation [26]. Moreover, advanced analytics and machine learning algorithms are increasingly being applied to predict complications such as sepsis, ventilator-associated pneumonia, or acute kidney injury, allowing for earlier interventions [27,28,29,30]. At the same time, digitalization supports quality improvement initiatives by enabling standardized data collection, benchmarking, and continuous performance monitoring [31,32].

However, the impact of ICU digitalization is not without challenges. Concerns regarding data privacy, system interoperability, and the risk of alert fatigue remain significant [33,34]. In addition, digital transformation requires substantial investment in infrastructure, training, and cultural adaptation among healthcare professionals [32,35,36]. Evaluating its effectiveness thus requires not only measuring clinical outcomes, but also considering safety, efficiency, and sustainability.

Even though digital health technologies are becoming more common in intensive care, evidence concerning their real-world implications for clinical outcomes and infection control remains unreliable and highly context-dependent. The majority of existing research focus on isolated digital tools or are undertaken in completely digitalized, high-resource settings, which limits their applicability to ICUs undertaking gradual digital transformation. Furthermore, data combining patient outcomes, infection epidemiology, and device utilization within the same cohort are limited, particularly in Eastern European healthcare systems. This study fills this gap by comparing consecutive trauma ICU admissions before and after unit-wide patient data management system (PDMS) adoption, providing practical data on how ICU digitalization may impact quality of care during standard clinical practice.

This study aimed to evaluate whether ICU digitalization through the implementation of aPDMS was associated with improvements in quality of care and patient safety, as reflected by length of stay, infection rates, device utilization, antibiotic exposure, and mortality among trauma ICU patients.

## 2. Materials and Methods

### 2.1. Study Design

This was a single-center, retrospective, before–after cohort study conducted in an adult ICU of a tertiary hospital. We compared two non-overlapping calendar periods: a pre-digitalization period (2021–2022) and a post-digitalization period (2025). The exposure of interest was the unit-level transition from predominantly paper-based documentation to a structured PDMS introduced in 2024. Clinical care protocols remained unchanged at the unit level; the intervention primarily modified the documentation workflow and data capture fidelity, including the standardization of medication prescriptions through pre-formatted templates with automatic body-weight adjustment, alongside structured nursing prescriptions and care bundles aimed at nosocomial infection prevention.

The study was designed and reported in accordance with the Strengthening the Reporting of Observational Studies in Epidemiology (STROBE) statement for observational cohort studies [37]. Period definitions, covariates, and sensitivity analyses were prespecified before inspection of between-period outcome differences.

### 2.2. Study Population

The study population comprised all consecutive Trauma ICU admissions recorded in the local ICU registry during the two periods. The total sample size was determined by the number of eligible cases available in the ICU registry, as no a priori power calculation was performed for this retrospective before–after analysis. Eligibility criteria included admissions with available core variables: age, sex, admission and discharge dates, ISS, Charlson comorbidity index, and ICU LOS. Exclusion criteria were admissions lacking these variables and records with missing discharge information, which precluded calculation of exposure time. Readmissions during the same hospitalization were retained as distinct episodes only if the registry recorded them as such. We applied no diagnostic or age restrictions beyond the requirement for the core variables. As the study was registry-based, no follow-up after ICU discharge was performed.

### 2.3. Digitalization

Digitalization refers to the implementation of an electronic PDMS used for point-of-care entry of observations and therapies, including vital signs, device placements, ventilation and antimicrobial days, and structured recording of nosocomial infections and microbiology results. The PDMS standardized data elements and timestamps, enabling consistent extraction of infection types and organisms and more complete capture of resource utilization. No new infection control bundles or antimicrobial stewardship policies were introduced contemporaneously with the PDMS; the change reflected documentation and data infrastructure rather than formal protocol changes.

Digitalization within the PDMS also encompassed standardized, pre-formatted order sets for both pharmacologic therapies and nursing care, including intensive care management prescriptions. Medication orders were automatically weight-adjusted and integrated with safety alerts, such as warnings for renal impairment when diuresis declined or serum creatinine increased, thereby supporting individualized dose adjustment. Nursing prescriptions and structured care plans were embedded directly in the system, facilitating infection-prevention practices and daily care workflows. Routine vital signs and device parameters automatically imported from bedside monitors, eliminating manual transcription. These features collectively reduced the clerical burden for physicians and nurses, allowing more direct patient care, while enhancing data consistency, traceability, and patient safety through automated notifications and standardized documentation. The PDMS collected physiologic and laboratory data in near real-time from network-connected monitors and ventilators through an integrated middleware system. Automated quality checks identified implausible or missing values, prompting manual verification. System logs were reviewed daily, and any data gaps > 15 min were reconciled using paper backups or device logs. Routine audits by the ICU IT team confirmed synchronization accuracy and overall data completeness exceeding 99%. Downtime events were infrequent and did not materially affect data integrity.

The PDMS was introduced in mid-2024 and reached full operational stability by the end of that year. All ICU staff completed structured training before 2025, and routine quality checks confirmed consistent data capture and system performance. The post-digitalization analysis (January–December 2025) therefore reflects a stable implementation phase without further software updates or workflow modifications that could bias outcome comparisons.

All patient data were stored on secure hospital servers within a restricted local network protected by multi-layer firewalls. Access to the PDMS required individual user authentication with role-based permissions for physicians, nurses, and administrators. Data transmission between bedside devices and the PDMS database was encrypted using hospital-approved secure communication protocols. Identifiable patient data were accessible only to authorized clinical personnel, while de-identified datasets were used for research analysis. The system complied with the EU General Data Protection Regulation (GDPR) and institutional IT security policies.

### 2.4. Data Sources and Variables

All data were abstracted from the ICU digital registry used for routine care and quality improvement. The dataset included demographics, baseline severity and comorbidity (age, sex, ISS, Charlson index), and admission date. Resource-use variables comprised ICU LOS (days), mechanical ventilation days, antibiotic days, and counts of device placements: central venous catheters (CVC), endotracheal intubations (ETT), and urinary catheters (UC). Clinical outcomes included ICU mortality and nosocomial infections recorded during the ICU stay. For infections, the registry provided the number of infection episodes and free-text fields for infection types and pathogens. For descriptive epidemiology, text fields were parsed with simple rules, splitting on common delimiters (commas, slashes, semicolons, “SI”/“and”) and normalizing tokens to uppercase. Device-days were not available in the registry, limiting calculation of standard device-associated infection rates.

### 2.5. Outcomes

The primary outcome was ICU length of stay. Secondary outcomes were ICU mortality; nosocomial infection burden summarized as the total number of episodes per cohort and as crude infections per 1000 ICU-days; mechanical ventilation and antibiotic exposure; device utilization (CVC, ETT, UC); and infection epidemiology, reported both as per-patient presence (whether a patient had at least one record of a given type or pathogen) and as mention-based distributions (the share of all recorded type or pathogen tokens within a period). We also summarized per-patient presence of major infection types, such as VAP, BSI, and UTI and key pathogens (*Acinetobacter* spp., *MRSA*, *Klebsiella* spp.). Quality of care was represented by quantitative indicators such as infection burden, length of stay, mortality, and resource use, which reflect safety and efficiency dimensions of ICU quality. No qualitative interviews or surveys were conducted.

### 2.6. Definitions

ICU LOS was defined as the interval in days from ICU admission to ICU discharge, consistent with registry computation. A nosocomial infection episode corresponded to an infection entry recorded by the ICU or infection-control team during the ICU stay. When multiple infection types or organisms were recorded for a single patient, all tokens were retained for the mention-based distributions; per-patient presence was defined as at least one token for that type or organism. Device variables were registry counts of placements per admission. Because device-days were unavailable, device-normalized infection indices (VAP per 100 ETT insertions) are interpreted as descriptive proxies rather than standard device-day rates.

### 2.7. Derived Metrics

We calculated the infection rate per 1000 ICU-days within each period as the number of infection episodes divided by the total ICU-days, multiplied by 1000. For infection epidemiology, we produced two complementary summaries: per-patient presence by period and mention-based distributions (the percentage of all recorded mentions within a period). For device-normalized indices, we expressed the number of patients with VAP, BSI, or UTI per 100 insertions of the corresponding device (ETT, CVC, UC), acknowledging the proxy nature of this measure in the absence of device-days.

### 2.8. Statistical Analysis

Continuous variables were summarized as median and interquartile range when distributions were skewed and as mean with standard deviation when approximately symmetric; categorical variables were summarized as counts and percentages. Between-period comparisons used the Mann–Whitney U test for continuous variables and the chi-square test or Fisher’s exact test, as appropriate, for categorical variables. For descriptive rate contrasts, we calculated crude Poisson rate ratios (RRs) with 95% confidence intervals (CI) based on episode counts and ICU-days. Multivariable models were prespecified with the study period (post vs. pre digitalization) as the exposure and adjustment for age, sex, ISS and Charlson comorbidity index. We fitted logistic regression for ICU mortality; Poisson regression with a log offset for ICU-days for nosocomial infection counts; Gamma regression with a log link for ICU length of stay; and negative binomial models with ICU-days offsets for ventilation-days and antibiotic-days. These models were estimated on complete cases for the adjustment covariates, and observations with non-positive ICU-days were excluded from rate-based models to ensure valid denominators.

We performed secondary analyses, such as adjusted logistic models for binary endpoints defined as any nosocomial infection and any antibiotic use and an unadjusted ICU length of stay comparison excluding stays shorter than 24 h. A Cox proportional hazards model for time to discharge alive, censoring deaths was also tested. In addition, we estimated overlap-weighted models using a propensity score including age, sex, ISS, Charlson index, and season to reduce covariate imbalance between periods for mortality, infection rate, and length of stay. For infection epidemiology, we complemented per-patient presence tests with chi-square or Fisher’s exact tests comparing the distribution of infection types and pathogens between periods. All sensitivity analyses were specified a priori to test the stability of the primary findings.

Analyses used complete-case observations for the prespecified covariates. No imputation was performed for outcomes or exposures. No imputation was performed for missing data, and all analyses were performed using two-sided tests with a significance threshold of *p* < 0.05. Statistical analyses were performed using IBM SPSS Statistics (version 27.0; IBM Corp., Armonk, NY, USA).

### 2.9. Bias

Potential bias was minimized by including all consecutive ICU admissions during each study period and by adjusting for major confounders (age, sex, ISS, comorbidity, season) in multivariable models. Observer bias was reduced by using registry-based, objective data rather than subjective assessment.

Study size was determined by the total number of eligible admissions during the defined periods, reflecting a complete census of available data rather than a priori sample-size calculation.

### 2.10. Ethics

The project used de-identified registry data collected as part of routine clinical care and ongoing quality improvement. The study protocol adhered to the Declaration of Helsinki and applicable national regulations [38]. Institutional approval was obtained from the ethics committee of the University of Medicine and Pharmacy Victor Babes Timisoara. (19/22 March 2022).

## 3. Results

### 3.1. Baseline Characteristics

A total of 108 patients were included, with 43 in the pre-digitalization period and 65 in the post-digitalization period. The two groups were comparable in terms of age (median 52.0 vs. 50.5 years, *p* = 0.73) and sex distribution (60.5% vs. 69.2% male, *p* = 0.46). Injury severity score (ISS) (34.0 vs. 34.0, *p* = 0.95), and comorbidity burden by the Charlson index (both median 1.0, *p* = 0.384), showed no significant differences between groups (Table 1).

However, ICU resource use parameters differed between periods. The median number of days on mechanical ventilation decreased from 9.0 (IQR 1.0–20.5) to 4.0 (IQR 1.0–10.0), although this reduction did not translate into statistical significance (*p* = 0.23). Similarly, antibiotic exposure tended to be shorter post-digitalization (median 5.0 vs. 5.0 days; IQR 0.0–8.5 vs. 0.0–16.5), with borderline significance (*p* = 0.05). Most notably, the ICU length of stay was significantly reduced, from 13.0 (IQR 3.0–24.5) to 6.0 (IQR 2.0–13.0) days (*p* = 0.02).

### 3.2. Clinical Outcomes and Resource Utilization

ICU mortality was numerically lower post-digitalization (18.6% vs. 6.2%), although this difference was not statistically significant (*p* = 0.06). Nosocomial infection rates were lower post-digitalization on a crude basis (30.8 vs. 42.2 per 1000 ICU-days; rate ratio 0.73, 95% CI 0.41–1.30; *p* = 0.28). Mechanical ventilation days and antibiotic exposure days trended to be lower post-digitalization, but no meaningful statistical difference was observed. (*p* = 0.23 and *p* = 0.05, respectively) (Table 2).

**Figure 1:**
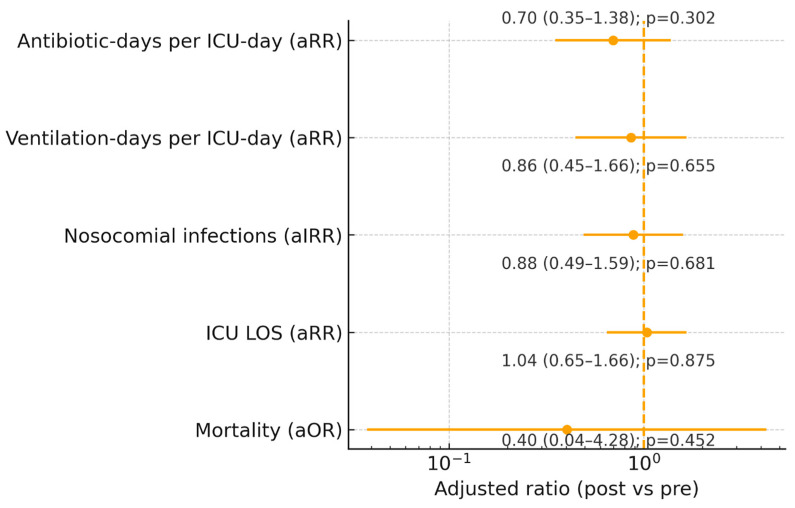
Forest plot of adjusted associations between the post-digitalization period (vs. pre) and study outcomes (from top to bottom: antibiotic-days per ICU-day, ventilation-days per ICU-day, nosocomial infections per ICU-day, ICU length of stay, and ICU mortality). Points denote adjusted ratios with 95% confidence intervals; the vertical dashed line indicates the null (ratio = 1). Models adjust for age, sex, ISS, Charlson index, and admission season; infection and resource-use outcomes include an offset for log (ICU-days).

After adjustment for age, sex, ISS, Charlson index, and admission season, there were no statistically significant differences between periods in ICU mortality (aOR 0.40, 95% CI 0.04–4.28; *p* = 0.452), nosocomial infection rates per ICU-day (aIRR 0.88, 95% CI 0.49–1.59; *p* = 0.681), ICU LOS (aRR 1.04, 95% CI 0.65–1.66; *p* = 0.875), ventilation-days per ICU-day (aRR 0.86, 95% CI 0.45–1.66; *p* = 0.655), or antibiotic-days per ICU-day (aRR 0.70, 95% CI 0.35–1.38; *p* = 0.302) (Table 3 and Figure 1).

### 3.3. Sensitivity Analyses

In logistic models for any nosocomial infection and any antibiotic use, adjusted associations remained non-significant (both *p* > 0.05). An additional sensitivity analysis excluding ICU stays shorter than 24 h yielded a similar reduction in length of stay (13.0 [3.0–24.5] vs. 6.0 [2.0–13.0] days; *p* = 0.021), consistent with the findings of the primary unadjusted comparison.

### 3.4. Infection and Epidemiology

The proportion of patients with ≥1 nosocomial infection decreased from 37.2% (pre-digitalization group) to 23.1% (post-digitalization group), with crude rates declining from 42.2 to 30.8 per 1000 ICU-days (rate ratio 0.73, 95% CI 0.41–1.30). These two indicators reflect different epidemiologic dimensions: the former measures infection occurrence per patient, whereas the latter adjusts for time at risk. Given the shorter post-implementation ICU stays, lower infection rates may partly reflect reduced exposure duration. The distribution of infection types did not change significantly overall (*p* for chi-square test = 0.26); however, per-patient analyses showed fewer ventilator-associated pneumonias (VAP) (−17.1 percentage points; *p* = 0.04) and bloodstream infection (BSI) (−15.6 percentage points; *p* = 0.04) in the post period, while urinary tract infection (UTI) was unchanged (*p* = 0.43). The pathogen mix showed a non-significant overall shift (*p* for chi-square test = 0.08); notably, *Acinetobacter* spp. was observed in 7 pre-period patients and none post-period (*p* for Fisher’s exact test = 0.001), whereas *Klebsiella* increased numerically (1 to 7 patients; *p* = 0.14) and Methicillin-Resistant *Staphylococcus aureus* (*MRSA*) was stable (6 to 6; *p* = 0.65) (Table 4).

### 3.5. Device Use

Device use remained common across both periods (Table 5). Total insertions increased post-digitalization for central venous catheters (CVC: 49 vs. 90), endotracheal tubes (ETT: 64 vs. 114), and urinary catheters (UC: 60 vs. 105). The proportion of patients with at least one CVC was similar between periods (72.1% vs. 67.7%; *p* = 0.78). ETT exposure was numerically lower post-digitalization (88.4% vs. 75.4%; *p* = 0.15). UC exposure showed a borderline decrease (93.0% vs. 78.5%; *p* = 0.05). These counts summarize device availability/placement; device-days were not available, so device-adjusted infection rates should be interpreted cautiously.

## 4. Discussion

The results of this study indicate that the introduction of a PDMS in the ICU was associated with favorable trends in key quality and safety indicators, including shorter length of stay and reduced crude infection burden. While adjusted analyses did not confirm statistically significant effects, the direction of change suggests potential clinical relevance. A major strength of digitalization lies in its capacity to integrate and standardize information from multiple sources [39,40]. By consolidating patient data in real time, clinicians are better equipped to recognize early warning signs of deterioration, enabling rapid interventions. Previous reports have shown that digital monitoring platforms and automated alerts can facilitate faster responses to sepsis, respiratory compromise, or arrhythmias, ultimately reducing preventable morbidity and mortality [41,42,43]. In our analysis, reductions in device-associated infections and more rational use of antibiotics reflect these improvements in decision-making and workflow. When interpreted separately, the infection metrics provide complementary but distinct insights. The reduced proportion of patients with at least one infection suggests that fewer individuals acquired infections during the digitalization period. In contrast, the lower infection rate per 1000 ICU-days should be viewed in the context of shorter ICU stays, which decrease exposure time and may partially explain the rate reduction. Together, these findings indicate possible progress in infection control practices but warrant cautious interpretation regarding true risk reduction.

In a prospective study implementing an EMR-embedded quality-control system, integrated with bedside monitors/ventilators, closed-loop medication scanning, scheduled assessment prompts, and real-time anomaly alerts, substantially improved documentation performance: median quality control time fell from 264 to 62 s, false vital-sign entries dropped from 9% to 1.33%, incomplete medication terminations from 3.33% to 1.67%, and missed assessment items from 8% to 1.33% [44]. A systematic review reported that digital tools consistently improved patient safety, reducing medication and procedural errors, enhancing compliance with ICU care bundles, improving triage accuracy, and shortening ICU length of stay [26].

Another relevant finding concerns device utilization, such as central venous catheters, invasive ventilation, and urinary catheters. Digital tracking of device days and automated reminders for timely removal have been shown to significantly lower the risk of device-associated complications [45,46,47]. Our results support this association, indicating that digital oversight may enhance infection control and shorten ICU stays without increasing staff workload.

Digitalization may also influence the relationship between antimicrobial management and ICU length of stay. In our cohort, antibiotic exposure tended to decrease following PDMS implementation, in parallel with a reduction in median ICU stay. These findings suggest that digital systems could promote more judicious antimicrobial use by improving documentation accuracy, enabling automated dose adjustments, and facilitating early identification of infection resolution. Previous studies have shown that electronic prescribing and clinical-decision-support tools are associated with reduced inappropriate antibiotic use and earlier de-escalation, which in turn can shorten hospitalization and limit antimicrobial resistance [14,15,48,49]. Although our adjusted analyses did not reach statistical significance, the concurrent downward trends in antibiotic-day and length-of-stay metrics indicate that digitalization may enhance the efficiency of infection management, supporting both patient recovery and antimicrobial stewardship.

In our setting, the PDMS primarily changed how clinical data and infection-prevention bundles were captured and acted upon in daily practice. Prior to digitalization, documentation relied on handwritten charts, manual transcription from monitors, and separate infection logs. After implementation, all bedside devices were integrated through the PDMS, enabling automated data import, time-stamped charting, and structured order sets for medication and nursing care. This led to greater completeness and legibility of records, but required extensive staff adaptation during the first months. The most immediate operational changes included reduced duplicate documentation, automated antibiotic dose adjustment by body weight, and real-time alerts for renal function decline or device removal reminders.

In addition to these operational changes, various well-known challenges can impede the transition from paper-based documentation to a digital PDMS, which may have an impact on the early-phase outcomes seen in our study. Digitalization frequently causes temporary workflow delays as employees acclimate to new interfaces, structured data fields, and changing documentation practices. Differences in digital literacy among team members, initial resistance to change, and the need for additional training sessions may all lead to inefficient or inconsistent data entry. Early usability issues, alert fatigue, and increased cognitive load during system adoption are all notable barriers. These limitations do not contradict the long-term benefits of PDMS deployment, but they serve to explain why the magnitude of improvement in clinical outcomes may be limited in the early post-digitalization era. The inclusion of these characteristics improves the interpretation of our data and indicates that the shift from paper to electronic medical records is a gradual process rather than an immediate driver of clinical improvement. Despite the reported improvements, several practical challenges emerged. Early in the rollout, inconsistent data entry and system downtime episodes occasionally limited data completeness. Moreover, not all parameters were captured digitally, which constrained our ability to calculate standardized infection rates. The neutral results in adjusted analyses may therefore reflect both the relatively small sample size and residual variability in data entry patterns during the first year of stable PDMS operation. Nonetheless, the observed trends toward shorter ICU stays and lower infection frequencies suggest that even incremental digital standardization can have tangible benefits for workflow efficiency and patient safety.

The impact of digitalization on mortality is more difficult to assess, as outcomes in critically ill patients are determined by a multitude of factors, including baseline severity of illness, comorbidities, and therapeutic strategies. Nonetheless, the trend toward improved survival observed in our study echoes findings from large-scale tele-ICU programs, which have demonstrated reduced mortality and length of stay when digital oversight is integrated into routine care [21,50]. While causality cannot be firmly established, it is reasonable to infer that better infection control, optimized device management, and timely interventions contribute to survival benefits. Bogale et al. highlighted that introducing electronic medical or health records was associated with a small but significant reduction in mortality: the pooled estimate across 42 studies in a systematic review showed a 3–7% decrease [51].

The study of Pankhurst et al. demonstrated that digitalization in the ICU through electronic chart implementation significantly reduced documentation errors and saved an average of 44 min of nursing time per patient per day, while improving data accuracy, accessibility, and user satisfaction, showing that EHR integration not only enhances efficiency but also strengthens patient safety and workflow continuity in critical care settings [52]. Similarly, other studies reported that electronic documentation reduces redundant form-filling, decreases paper use, and lowers operational costs, enabling medical staff to dedicate more time to direct patient care and contributing to both workflow efficiency and environmental sustainability [53,54]. Schol et al. further showed that digitalization of ICU diaries improves accessibility, facilitates daily integration into workflows, and enhances staff engagement through structured feedback systems [55]. On the contrary, a review across 61 studies showed that digital hospitals improved clinician satisfaction and information access but brought heavier documentation, strained bedside communication, mixed effects on safety and care delivery, and only limited, mostly neutral-to-positive patient satisfaction evidence [56]. Beyond critical care, the principles of data integration and individualized monitoring promoted by ICU digitalization align with broader efforts to optimize metabolic and systemic health [57,58,59,60]. A multicenter cross-sectional study from the Netherlands explored ICU professionals’ perspectives on implementing and using a digital intensive care unit diary, highlighting key facilitators such as user-friendly access, enthusiastic champions, comprehensive information, and privacy assurance, underscoring that successful digitalization in the ICU depends not only on technology adoption but also on staff engagement, education, and organizational support [61]. Despite these encouraging results, challenges persist, including alert fatigue, data security, privacy risks, and unequal IT resources across institutions. The heterogeneity of ICU infrastructure further complicates standardization, reinforcing that sustained training, usability testing, and leadership support are essential for realizing the full benefits of digitalization in critical care.

### Limitations

This study has several limitations that should be acknowledged. First, the sample size was modest, reflecting a single-center design limited to trauma ICU admissions. Second, the registry did not include device-day denominators, which restricted the computation of standardized device-associated infection rates. Third, although the PDMS improved documentation accuracy, some early variability in data entry practices may have influenced data completeness during the first year of full operation. Fourth, the temporal gap between the pre-digitalization (2021–2022) and post-digitalization (2025) periods introduces potential temporal bias, as unmeasured changes in case mix, staffing, or infection-control practices may have occurred during the intervening years. Such progressive, system-level evolutions, which occur in any clinical context, may have an impact on outcomes independent of PDMS deployment. Additionally, unmeasured secular trends may have influenced the results. Although no formal changes in infection-prevention protocols, antimicrobial stewardship policies, or staffing occurred between 2021 and 2022 and 2025, subtle shifts in patient case mix, workflow efficiency, or institutional focus on infection control could have contributed to the observed trends. The absence of statistical significance in multiple adjusted models, along with large confidence ranges, hinders causal inference. These findings thus represent indicative trends rather than conclusive outcomes of PDMS implementation. The absence of statistically significant effects is most likely due to a combination of small sample size, early-stage digital deployment, and the inherently multivariate character of ICU performance measurements. Overall, the study should be viewed as exploratory. Digitalization may have an indirect impact, through increased workflow uniformity, documentation completeness, and decision support, but multicenter studies with larger cohorts and longer follow-up are necessary to discover substantial therapeutic advantages.

## 5. Conclusions

In this single-center, cohort study ICU evaluation of PDMS digitalization, we examined clinical outcomes, infection epidemiology, and device use. Post-implementation, ICU stay and crude infection burden were lower, with fewer VAP/BSI and no *Acinetobacter* detections, while *Klebsiella* increased; device use remained common with more total insertions recorded. After adjustment for age, sex, ISS, Charlson, and season, differences in mortality, LOS, and infection/resource rates were not statistically significant. Although adjusted analyses showed no statistically significant associations, the overall findings support the feasibility and safety of ICU digitalization and its potential to enhance process quality and data integrity as the system matures. From a health-policy perspective, these findings emphasize the importance of supporting structured digitization in ICUs, including investments in PDMS infrastructure and defined data procedures. Strengthening national frameworks for digital integration may improve surveillance, quality monitoring, and long-term critical-care outcomes. Larger multicenter studies with device-day denominators and standardized definitions are needed to establish causal impact.

## 6. Implications for Clinical Practice

The results of this study have practical relevance for critical care practice. Even in the lack of statistically significant adjusted effects, the reported reductions in unadjusted ICU length of stay, selected device-associated infections, and crude infection burden indicate that structured digitalization via PDMS can enable safer and more efficient ICU operations. Standardized documentation, automated data collecting from bedside devices, and integrated clinical decision support may lead to earlier detection of complications, enhanced infection prevention procedures, and better antibiotic stewardship. From a clinical standpoint, these outcomes suggest that PDMS deployment can be advantageous even in ICUs undergoing progressive digital transformation, without necessitating urgent protocol revisions or sophisticated predictive analytics. This encourages the use of PDMS platforms by clinicians and hospital managers as a basis for quality enhancement, monitoring, and the eventual integration of advanced decision-support technologies.

## 7. Implications for Future Research

Larger sample sizes and multicenter studies are required to validate the trends found and to more thoroughly evaluate any possible cause-and-effect connections between PDMS implementation and clinical outcomes. To more precisely measure infection risk and resource use, future research should use longitudinal designs, time-to-event analyses, and consistent device-day denominators. Furthermore, it may be possible to distinguish the effects of digital infrastructure from concurrent organizational or clinical practice changes by conducting comparative research across institutions at various stages of digital maturity. Since these technologies have the potential to enhance risk stratification, early complication diagnosis, and individualized treatment plans, future research should also examine the integration of artificial intelligence and predictive analytics into ICU digital platforms. However, thorough external validation and ethical supervision are necessary for their clinical application. To make sure that technology enhances rather than replaces professional judgment in critical care settings, ongoing assessment of digital treatments is crucial.

## Figures and Tables

**Table 1:** Baseline characteristics and resource-use parameters of patients admitted to the ICU during the pre- and post-digitalization periods.

Variable	Pre-Digitalization (*n* = 43)	Post-Digitalization (*n* = 65)	*p*-Value
Age, years—median (IQR)	52.0 (33.5–63.0)	50.5 (28.8–64.0)	0.73
Male, *n* (%)	26 (60.5%)	45 (69.2%)	0.46
ISS—median (IQR)	34.0 (25.0–45.0)	34.0 (23.0–48.0)	0.95
Charlson—median (IQR)	1.0 (0.0–2.0)	1.0 (0.0–2.0)	0.38
Mechanical ventilation, days—median (IQR)	9.0 (1.0–20.5)	4.0 (1.0–10.0)	0.23
Antibiotic exposure, days—median (IQR)	5.0 (0.0–16.5)	5.0 (0.0–8.5)	0.05
ICU length of stay, days—median (IQR)	13.0 (3.0–24.5)	6.0 (2.0–13.0)	0.02 *

Legend: * *p* < 0.05.

**Table 2:** Unadjusted outcomes comparing pre- and post-digitalization cohorts.

Outcome	Pre-Digitalization (*n* = 43)	Post-Digitalization (*n* = 65)	*p*-Value
ICU mortality, *n* (%)	8 (18.6%)	4 (6.2%)	0.06
ICU length of stay, days—median (IQR)	13.0 (3.0–24.5)	6.0 (2.0–13.0)	0.02 *
Mechanical ventilation, days—median (IQR)	9.0 (1.0–20.5)	4.0 (1.0–10.0)	0.23
Antibiotic exposure, days—median (IQR)	5.0 (0.0–16.5)	5.0 (0.0–8.5)	0.05
Nosocomial infection rate—per 1000 ICU-days (counts; RR)	42.2 (28/664 days)	30.8 (20/650 days)	0.28 (RR 0.73, 95% CI 0.41–1.30)

Legend: * *p* < 0.05.

**Table 3:** Adjusted associations (post vs. pre) from multivariable models including age, sex, ISS, Charlson index, and admission season. Infection and resource-use models include log (ICU-days) as an offset.

Outcome	Adjusted Effect (Post vs. Pre)	*p*-Value
Mortality	aOR 0.40 (95% CI 0.04–4.28)	0.45
Nosocomial infections per ICU-day	aIRR 0.88 (95% CI 0.49–1.59)	0.68
ICU length of stay	aRR 1.04 (95% CI 0.65–1.66)	0.87
Ventilation-days per ICU-day	aRR 0.86 (95% CI 0.45–1.66)	0.65
Antibiotic-days per ICU-day	aRR 0.70 (95% CI 0.35–1.38)	0.30

**Table 4:** Nosocomial infection epidemiology by study period. Section A summarizes burden (patients, ≥1 infection, total episodes, ICU-days, and crude infection rate per 1000 ICU-days with Poisson RR and 95% CI). Section B shows per-patient presence of infection types with *p*-values from chi-square test or Fisher’s exact tests. Section C shows per-patient presence of selected pathogens.

Section	Metric/Item	Pre-Digitalization	Post-Digitalization	*p*-Value/Effect
A. Infection burden	Patients (*n*)	43	65	
	Any infection, *n* (%)	16 (37.2%)	15 (23.1%)	
	Total infection episodes, *n*	28	20	
	ICU-days, total	664	650	
	Episodes per 1000 ICU-days	42.2	30.8	RR 0.73 (95% CI 0.41–1.30)
B. Infection types (per-patient)	VAP	12 (27.9%)	7 (10.8%)	0.04 *
B. Infection types (per-patient)	BSI	10 (23.3%)	5 (7.7%)	0.04 *
B. Infection types (per-patient)	UTI	4 (9.3%)	3 (4.6%)	0.43
B. Infection types (per-patient)	Wound infection	0 (0.0%)	2 (3.1%)	0.51
B. Infection types (per-patient)	Enterocolitis	0 (0.0%)	1 (1.5%)	1
C. Pathogens (per-patient)	*Acinetobacter* spp.	7 (16.3%)	0 (0.0%)	0.001 **
C. Pathogens (per-patient)	*MRSA*	6 (14.0%)	6 (9.2%)	0.65
C. Pathogens (per-patient)	*Klebsiella* spp.	1 (2.3%)	9 (13.8%)	0.04 *

Abbreviations: BSI-bloodstream infection; MRSA-Methicillin-Resistant *Staphylococcus aureus*; UTI-urinary tract infection; VAP-ventilator-associated pneumonia. Legend: * *p* < 0.05; ** *p* < 0.001.

**Table 5:** Device utilization by period. Values shown are total insertions and the number (%) of patients with ≥1 device.

Device	Total Insertions (Pre)	Total Insertions (Post)	Patients with Device (Pre)	Patients with Device (Post)	*p*-Value
CVC	49	90	31/43 (72.1%)	44/65 (67.7%)	0.78
ETT	64	114	38/43 (88.4%)	49/65 (75.4%)	0.15
UC	60	105	40/43 (93.0%)	51/65 (78.5%)	0.05

Abbreviations: CVC-central venous catheter; ETT-endotracheal intubation; UC-urinary catheter.

## Data Availability

Data available from the corresponding author.
